# Phosphomannopentaose sulfate (PI-88) suppresses angiogenesis by downregulating heparanase and vascular endothelial growth factor in an oxygen-induced retinal neovascularization animal model

**Published:** 2012-06-20

**Authors:** Xian-Jun Liang, Ling Yuan, Jie Hu, Hong-Hua Yu, Tao Li, Shao-Fen Lin, Shi-Bo Tang

**Affiliations:** 1 The State Key Laboratory of Ophthalmology Zhongshan Ophthalmic Center, Sun Yat-Sen University, Guangzhou, China; 2 Foshan Hospital of Traditional Chinese Medicine, Foshan, Guangdong, China; 3 The First Affilliated Hospital of Kunming Medical College, Kunming, Yuannan, China

## Abstract

**Purpose:**

Vascular endothelial growth factor (VEGF) is the most potent angiogenic mitogen, and has been associated with angiogenesis. Heparanase is an endoglycosidase that specifically cleaves heparan sulfate side chains, which can induce VEGF expression. The aims of the present study were to evaluate the heparanase expression and its relationship with VEGF in the retina of oxygen-induced retinopathy (OIR) mice, and to investigate the effect of the heparanase inhibitor phosphomannopentaose sulfate (PI-88) in the OIR retinas.

**Methods:**

Seventy-seven newborn C57BL/6 mice were involved in this study. On postnatal day 7 (P7), pups were exposed to a hyperoxia condition (75% oxygen) for 5 days, and on P12, the mice were returned to room air. Control mice were exposed to room air from birth until P17, with normally developing retinal vasculature. PI-88 was administered intraperitoneally to OIR mice at a dose of 35.7 mg/kg/day for 5 consecutive days. The expression level of heparanase and VEGF in the retinas was assayed using immunohistochemistry, Q-RT–PCR, and western blot.

**Results:**

The expression levels of heparanase and VEGF were increased in the OIR retinas compared with the control mice. The Q-RT–PCR results showed that the mRNA expression levels of heparanase and *VEGF* in OIR retina were increased 1.71 fold (p<0.0001) and 4.34 fold (p<0.0001), respectively. The western blot results showed that the protein expression levels of heparanase and VEGF were increased 1.49 fold (p<0.0001) and 1.72 fold (p<0.0001), respectively, in the OIR retinas compared with the normal retinas. The immunohistochemistry analysis revealed that the heparanase and VEGF signals were intense in the retinal vascular endothelia of the OIR mice but faint in those of the normal controls. The increased protein and mRNA expression levels of heparanase and VEGF in the mouse retinas were significantly decreased by PI-88 administration (p<0.0001).

**Conclusions:**

Heparanase expression was upregulated and correlated with an increase in VEGF expression in the OIR mouse retinas, and might be involved in the progress of retinopathy of prematurity. Inhibition of heparanase expression by PI-88 could be used as a novel therapeutic method for retinopathy of prematurity.

## Introduction

Retinopathy of prematurity (ROP) is one of the leading causes of blindness in children worldwide, although considerable progress has recently been made in surgical strategies [1]. It is estimated that each year, 3,400 infants suffer from ROP-related visual impairments and 650 will become blind [2,3]. ROP is characterized by pathological ocular angiogenesis or retinal neovascularization (NV), mostly by exposure of low-birthweight infants to hyperbaric oxygen to maintain arterial oxygen tension in an appropriate range [4]. Nevertheless, the specific mechanism leading to ROP remains unknown and is difficult to study in human infants. Many molecules are involved in angiogenesis, of which vascular endothelial growth factor (VEGF) is one of the best studied. VEGF stimulates the elongation of epithelial cells and subsequently can induce the formation of new vessels [5].

Heparanase is an endoglycosidase that degrades heparan sulfate (HS) in the extracellular matrix (ECM) and cell surface, and plays significant roles in cancer metastasis and angiogenesis [6]. Heparan sulfate proteoglycans (HSPGs) are macromolecules consisting of a protein core to which HS chains are linked [7]. HSPGs are present on the membranes of eukaryotic cells (i.e., syndecans and glypicans) or as molecules that are secreted into the ECM (i.e., perlecans). HS chains bind a large variety of molecules, such as ECM structural proteins, growth factors, chemokines, and enzymes, thereby participating in central biologic processes, including adhesion, proliferation, survival, and differentiation [7,8]. Therefore, cleavage of HS side chains is expected to not only alter the integrity of the extracellular matrix but also release HS-bound growth factors, chemokines, and cytokines. Meanwhile, HS fragments can modulate the activities of growth factors such as basic fibroblast growth factor and VEGF [9–11]. In addition, heparanase is actively involved in regulating *VEGF* gene expression via c-Src activation to promote angiogenesis [12].

Phosphomannopentaose sulfate (PI-88) is a synthetic polysulfated oligosaccharide that inhibits the activity of the extracellular matrix–degrading enzyme heparanase [13]. PI-88 is a 2-kDa heparan sulfate mimetic structurally distinct from heparin, which is a heterogeneous mixture of polysaccharide chains of varying lengths with molecular weights of 3 to 30 kDa. PI-88 was primarily developed as an antitumor agent and can potently inhibit tumor metastasis and angiogenesis [13–16]. Additionally, human and mammalian toxicology studies have demonstrated that PI-88 is well tolerated and has low anticoagulant activity [17]. PI-88 is currently in Phase II and III clinical trials to assess its therapeutic potential as an anticancer drug [14,18].

However, it is unknown whether heparanase is involved in ROP and contributes to angiogenesis, whether heparanase expression is closely related to VEGF expression in ROP, and whether heparanase could become a new therapeutic target for ROP. In this study, we investigated an oxygen-induced retinopathy (OIR) mouse model to try to answer these questions.

## Methods

### Induction of oxygen-induced retinopathy and treatment

The studies were approved by the animal care and use committee of Zhongshan Ophthalmic Center, Sun Yat-Sen University. We adopted the standard OIR model as described previously [19]. Seventy-seven specific pathogen free (SPF) newborn C57BL/6 mice were obtained from the Traditional Chinese Medicine Center for Animals of Guangzhou University. Food, water, light, temperature, and other conditions were the same for all animals. These mice were randomly divided into three groups: Group A, the control group (normal mice, n=25), was reared in the standard SPF level for the duration of the experiment. Group B was the OIR model group (OIR mice, n=26). Group C was OIR mice receiving PI-88 (Progen Industries Limited, Brisbane, Australia) (dissolved in sterile phosphate-buffered saline, PBS, pH 7.4) at a dose of 37.5 mg/kg/d intraperitoneally for 5 consecutive days (OIR mice+PI-88, n=26).

### Mouse model of oxygen-induced retinopathy and treatment

The OIR mouse model was established as described previously [19]. Briefly, Group B and Group C mice were placed with their nursing mothers in the same covered plastic box with 75% oxygen from P7 (postnatal day 7) through P12. The oxygen was delivered at 75±2% and was checked at least three times daily during the oxygen exposure period. The oxygen concentration was measured with an Oxygen Monitor (BioSpherix, Ltd., New York, NY). On P12, the animals were returned to room air for 5 days (P17). The Group C mice were injected with PI-88 at a dose of 35.7 mg/kg/day intraperitoneally for 5 consecutive days. Finally, all 77 mice were euthanized using a lethal intraperitoneal injection of chloral hydrate (360 mg/kg) at P17. The retinal tissues of all 77 mice were used for immunohistochemistry, Q-RT–PCR, and western blotting.

### Fluorescein isothiocyanate–dextran (FITC-Dextran) perfusion of the retinal blood vessels

To study the retinal vascular pattern, systemic perfusion was performed using FITC-dextran (Sigma, St. Louis, MO) and polyphosphate-L-lysine (Sigma) in paraformaldehyde (PFA; Sigma). The animals were given a dose of 3 ml/kg chloral hydrate for anesthesia, and a median sternotomy was performed. The left ventricle of the heart was identified and perfused with 0.5 ml 4% PFA (50 mg/ml FITC-dextran and 10 mg/ml polyphosphate-L-lysine in 4% PFA) using a 1-ml tuberculin syringe with a 26-gauge needle for less than 30 s, until a yellowing of the liver was observed. The eyes of each mouse were then enucleated. Eyes were placed in 4% PFA for 30 min. Under a dissecting microscope (Axioplan2, ZEISS, Inc., Oberkochen, Germany), the retina was removed and flat-mounted by making radial cuts. Each retina was imaged with a fluorescence microscope (Axiovert100, ZEISS, Inc.).

### Immunohistochemistry staining for heparanase and vascular endothelial growth factor in oxygen-induced retinopathy retinas

Immunohistochemistry was performed as previously described, with minor modifications [20]. Deparaffinized retinal sections were blocked in 3% hydrogen peroxide and 5% BSA (Sigma). Retinal sections were incubated overnight at 4 °C with polyclonal rabbit anti-rat heparanase-1 antibody (1:1,000; Abcam, Cambridge, MA) and VEGF antibody (1:1,000; Abcam). After being washed with PBS 3 times, sections were incubated with horseradish peroxidase–conjugated goat antirabbit immunoglobulin G (Invitrogen, Carlsbad, CA). Color was developed using a 3,3′-diaminobenzidine kit (Vector Laboratories, Burlingame, CA) for 5 min, followed by counterstaining with Mayer’s hematoxylin. Immunostaining intensity was quantitatively analyzed using Image-Pro Plus software (version 5.1 for Windows; Media Cybernetics, Bethesda, MD) [21,22].

### Real-time polymerase chain reaction analysis for the expression of heparanase and vascular endothelial growth factor in oxygen-induced retinopathy retinas

We extracted total RNA from retinas harvested from the different groups using TRIzol (Invitrogen). The concentration and purity of the RNA were determined with a spectrophotometer. The RNA was purified using Amplification Grade DNase I (AMPD1; Sigma), and reverse transcriptase (PrimeScript RT reagent Kit, DRR037A; TaKaRa, Shiga, Japan) was used to synthesize cDNA. Q-RT–PCR assays were performed with SYBR Green Real-Time PCR Master Mix (SYBR Premix Ex Taq kit; TaKaRa), and gene expression was measured using SYBR green II (Qiagen, Hilden, Germany) with the ABI PRISM7000 real-time (Life Technologies Co., Ltd., Carlsbad, CA) PCR system. The PCR primers used to detect *VEGF*, heparanase, and *18S* rRNA were as follows: heparanase sense strand: 5′-CTC GAA GAA AGA CGG CTA AGA-3′, and reverse strand: 5′-TGG TAG CAG TCC GTC CAT T-3′; *VEGF* sense strand: 5′-AAG GAG AGC AGA AGT CCC ATG A-3′, and reverse strand: 5′-CAC AGG ACG GCT TGA AGA TGT-3′; *18S* rRNA sense strand: 5′-TTC CGA TAA CGA ACG AGA CTC T-3′ and reverse strand: 5′-TGG CTG ACA CGC CAC TTG TC-3′.

### Western blot assay for the expression of heparanase and vascular endothelial growth factor in oxygen-induced retinopathy retinas

Harvested retinas were homogenized in 150 µl lysis buffer containing 50 mM Tris-HCl (pH 6.8), 150 mM NaCl, 0.5% Triton X-100, and 10 µl protease inhibitor cocktail (Sigma-Aldrich, Inc., St. Louis, MO), and then centrifuged at 15,000× g at 4 °C for 30 min. Supernatants were collected and mixed with 5× sample loading buffer to prepare samples. Samples were electrophoresed on 12% sodium dodecyl sulfate gels and subsequently transferred to polyvinylidene difluoride membranes (Bio-Rad, Hercules, CA). Each membrane was blocked with 5% skim milk in Tris-buffered saline containing 0.5% Tween-20 at room temperature for 1 h. Next, the membranes were incubated with polyclonal rabbit antihuman heparanase-1 antibody (1:1,000) and polyclonal rat antihuman β-actin antibody (1:1,000; Abcam) at 4 °C overnight. The heparanase-1 antibody recognizes primarily the 50 kDa active form of the enzyme. The β-actin antibody recognizes primarily the 42 kDa active form of the enzyme. The membranes were then incubated with a secondary antibody at room temperature for 1 h. Finally, the blots were developed by a chemiluminescence kit (Cell Signaling Technology Inc., Danvers, MA). Semiquantitative analysis was performed by measuring the optical densities of the bands.

### Statistical analysis

Data are presented as mean±standard deviation (SD). Statistical significance was analyzed with one-way ANOVA using GraphPad Prism 4.0 software system (GraphPad, San Diego, CA) and the statistical software program SPSS 16.0 for Windows (Chicago, IL). P values of <0.05 were considered significant in all cases.

## Results

### Fluorescein isothiocyanate-Dextran systemic perfusion

**Figure 1:**
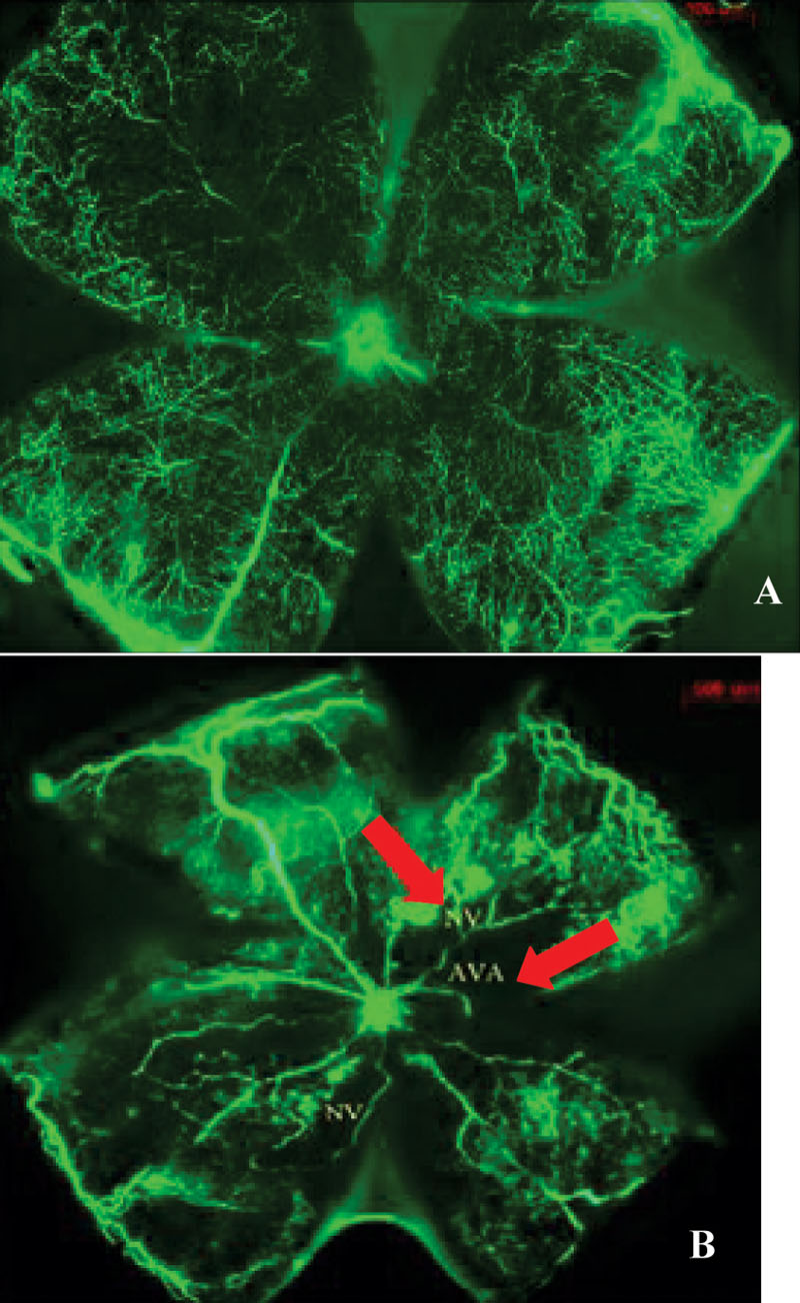
Fluorescein isothiocyanate-dextran (FITC) perfusion of the retinal blood vessels was observed under a fluorescent microscope in postnatal day 17 (P17) oxygen-induced retinopathy (OIR) retinas. **A**: Mice were perfused with FITC-labeled fluorescein sodium in normal oxygen pressure. **B**: Mice were perfused with FITC-labeled fluorescein sodium in hypoxia. Fluorescence images showing retinal vessels were tortuous and expanded in volume. Capillary hemangioma was observed, and retinal vascular morphology exhibited abnormal distribution at the junction of the perfused area and non-infused area. Significant expansion of large vessels, large avascular area, and extensive angiogenesis were observed. There are non-vascular area and neovascular tuffs in oxygen-induced retinopathy (OIR) mice (arrow).

To determine whether the OIR mouse model was successfully established, FITC-dextran perfusion of the retinal blood vessels was observed under a fluorescent microscope in P17 OIR retinas. As shown in Figure 1A, fluorescence images showing retinal vessels were tortuous and expanded in volume. Capillary hemangioma was ob-served, and retinal vascular morphology exhibited abnormal distribution at the junction of the perfused area and non-infused area. Significant expansion of large vessels, large avascular area, and extensive angiogenesis were observed. Thus, the OIR model was successfully established (Figure 1B).

### Immunohistochemistry staining analyses

**Figure 2:**
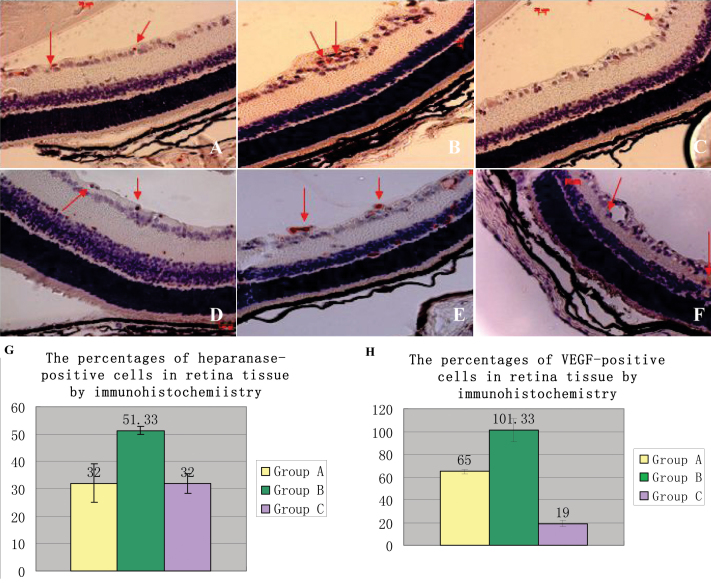
The effects of Immunohistochemistry staining for retina sections were accessed in this study. **A**-**C**: The retina sections stained for heparanase positive cells were show. **A**: It shows a normal mouse retina section on postnatal day 17 (P17). **B**: It displays the retina section in oxygen-induced retinopathy (OIR) mice. **C**: It shows PI-88 treatment of an OIR mouse retina. In all of the sections, the red arrow points to heparanase positive cells. **D**-**F**: The retina sections stained for vascular endothelial growth factor (VEGF) positive cells were show. **D**: It shows a normal mouse retina section on P17. **E**: It displays the retina section in OIR mice; F: It shows phosphomannopentaose sulfate (PI-88) treatment of an OIR mice retina. In all of the sections, the red arrow points to VEGF positive cells. **G**: The percentages of heparanase-positive cells in retina tissue by immunohistochemistry were as follows: The percentage of Group A was 32.00±7.00; the percentage of Group B was 51.33±1.53; the percentage Group C was 32.00±3.61. **H**: The percentages of VEGF-positive cells in retina tissue by immunohistochemistry were as follows: The percentage of Group A was 65.00±2.00. The percentage of Group B was 101.33±10.11; The percentage Group C was 19.00±2.65. The differences among groups were significant (p<0.0001).

In the present study, the expression levels of heparanase and VEGF were increased in OIR retinal vascular endothelial cells (Figure 2B,E). Meanwhile, the expression levels of heparanase and VEGF were decreased in response to PI-88 treatment. Positive cells were stained red, and cytoplasmic staining was clearly observed in the retinal cells (Figure 2C,F). As shown in Figure 2B-F, the staining for heparanase and VEGF was intense in the endothelia of blood vessels in the OIR retinas but faint in those of normal retinas and OIR+PI-88 retinas.

Quantitative analysis of immunostaining fluorescence intensity showed that in vascular endothelial cells, the heparanase expression was increased in the OIR retinas compared with the normal retinas (p=0.003), and decreased in the PI-88-treated OIR retinas compared with the OIR retinas (p=0.001; Figure 2G). In vascular endothelial cells, the VEGF expression was significantly increased in the retinas of Group B compared with those of Group A (p<0.0001) and decreased in Group C compared with Group B (p=0.001; Figure 2H).

### Real-time polymerase chain reaction analysis

**Figure 3:**
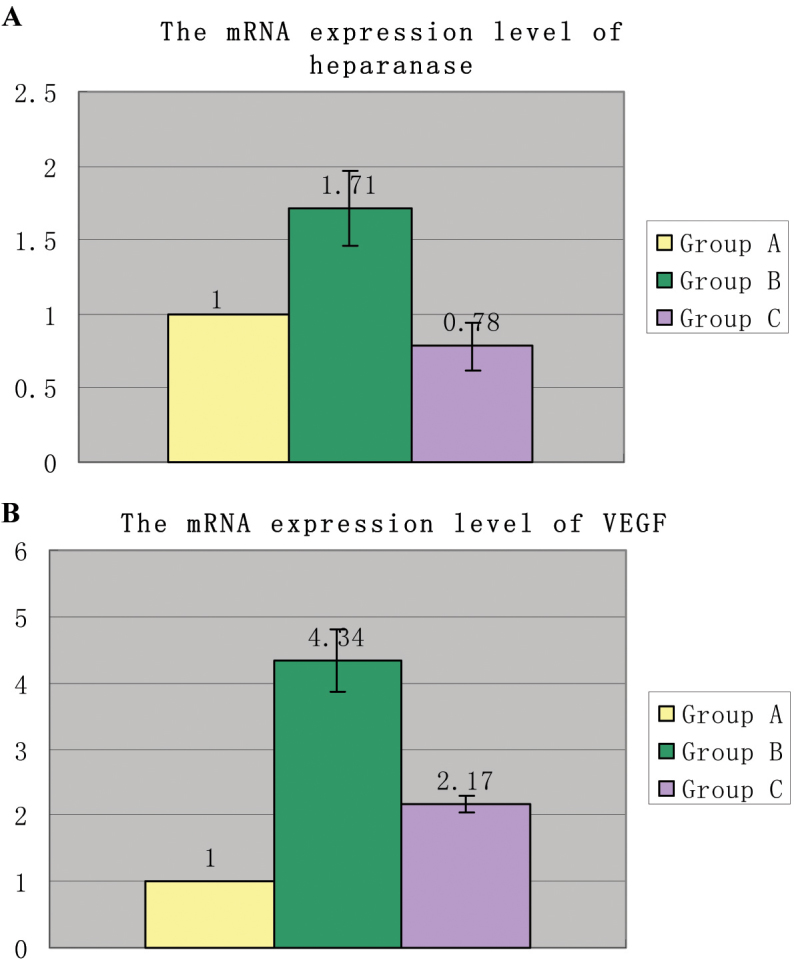
The effects of heparanase and vascular endothelial growth factor (VEGF) mRNA expression levels on oxygen-induced retinopathy (OIR) mice and normal mice were as follows. **A**: The mRNA expression level of heparanase increased 1.71 fold in Group B compared with Group A (p<0.0001) but decreased 2.18 fold in PI-88–treated mice compared with the group B (p<0.0001). There were significant differences in heparanase expression among the three groups (p<0.0001). **B**: The mRNA expression level of vascular endothelial growth factor (*VEGF*) increased 4.34 fold in Group B compared with Group A (p<0.0001) but decreased 2.00 fold in phosphomannopentaose sulfate (PI-88) treated mice compared with Group B (p<0.0001). There were significant differences in VEGF expression among the three groups (p<0.0001).

We used real-time PCR to determine if the mRNA expression levels of heparanase and *VEGF* were changed in retinal vascular endothelial cells in OIR mice. The results indicated that the expression levels of heparanase and *VEGF* mRNAs increased in the OIR mice compared with Group A and Group C. In contrast, the expression of heparanase and *VEGF* was decreased by treatment with PI-88 (Group C). As shown in Figure 3A, the mRNA expression level of heparanase increased 1.71 fold in Group B compared with Group A (p<0.0001), and the mRNA expression level of heparanase decreased 2.18 fold in Group C compared with Group B (p<0.0001). There were significant differences in the heparanase expression level among the three groups (p<0.0001). Compared with Group A, the mRNA expression level of *VEGF* increased 4.34 fold in Group B (p<0.0001), Compared with Group B, the mRNA expression level of *VEGF* decreased 2.00 fold in Group C (p<0.0001). There were significant differences in the *VEGF* expression level among the three groups (p<0.0001; Figure 3B).

### Western blotting

**Figure 4:**
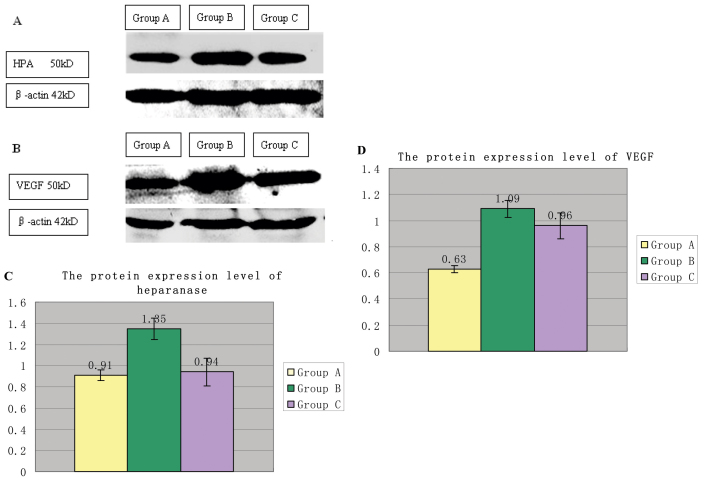
The effects of heparanase and vascular endothelial growth factor (VEGF) protein expression levels on three group mice were as follows. **A**: It shows the expression of heparanase in the three groups by western blot. The band that represents the 50 kDa activated enzyme was more intense in Group B than in Group A, and the level was decreased by phosphomannopentaose sulfate (PI-88) treatment. **B**: It shows the expression of VEGF in the three groups by western blot. The band that represents the 50 kDa activated enzyme was more intense in Group B than in Group A, and the level was decreased by PI-88 treatment. **C**: The protein expression level of Group B increased compared with Group A. There was a significant difference in the heparanase expression between the groups (p<0.0001). **D**: The protein expression level in Group B increased compared with Group A. There was a significant difference in the VEGF expression between the groups (p<0.0001).

We used western blotting to investigate if the protein expression levels of heparanase and VEGF changed in the retinal vascular endothelial cells in the OIR mice. Western blot analysis for heparanase and VEGF demonstrated that the protein expression levels of heparanase and VEGF in the OIR mice increased (Figure 4A,B). Compared with Group A, the protein expression level of heparanase increased 1.49 fold in Group B (p=0.000), Compared with Group B, the protein expression level of heparanase decreased 1.44 fold in Group C (p=0.000). There were significant differences in the heparanase protein expression among the three groups (p<0.0001; Figure 4C). Additionally, compared with Group A, the protein expression level of VEGF increased 1.72 fold in Group B (p<0.0001). Compared with Group B, the protein expression level of VEGF decreased 1.14 fold in Group C (p<0.0001). There were significant differences in the VEGF protein expression among the three groups (p<0.0001; Figure 4D).

## Discussion

In the present study, we demonstrated that heparanase expression was significantly increased in the retinal vascular endothelia of OIR mice but there was faint staining in normal mice. Heparanase is an endoglycosidase that degrades heparan sulfate glycosaminoglycan, leading to the loss of basement membrane integrity and the release of heparan sulfate–bound angiogenic and growth-promoting factors [23], which subsequently stimulate tumor blood vessel growth, cellular invasion, migration, adhesion, metastasis, differentiation, and proliferation [24–26]. Heparanase is overexpressed in a variety of tumors [27–32], corneal neovascularization [33], and the diabetic rat retina [34]. These findings suggest that heparanase expression is active in the vessel endothelium of newborn mouse retinas impaired by hyperoxia. Endothelial cells are an important cellular source of heparanase enzymatic activity [35–37].

Along with the high expression of heparanase, the expression of VEGF was also enhanced in OIR mice, as previously reported. Zetser et al. [12] reported that heparanase is actively involved in regulating *VEGF* gene expression. Heparanase expression is upregulated and is associated with increased VEGF expression in other retinopathy models, such as diabetic rats [34]. A novel mechanistic pathway driven by heparanase expression in myeloma cells can induce elevated levels of VEGF and shedding of syndecan-1 from matrix-anchored complexes that together activate integrin and VEGF receptors on adjacent endothelial cells, thereby stimulating tumor angiogenesis [38]. Cohen-Kaplan et al. [39] demonstrated that heparanase overexpression by epidermoid, breast, melanoma, and prostate carcinoma cells can induce a three- to fivefold elevation in VEGF C expression in vitro, and heparanase gene silencing was associated with decreased VEGF C levels. Heparanase also seems to be actively involved in regulating *VEGF* gene expression via c-Src activation to promote angiogenesis [9]. Our results showed that the protein and mRNA expression levels of heparanase and VEGF were significantly increased in OIR retinas. These findings suggest that heparanase expression is upregulated by oxygen overexposure and has a close relationship with the VEGF level in OIR retinal vascular endothelial cells. Therefore, heparanase may be involved in the angiogenic responses of OIR mice via promoting VEGF expression.

Our study showed that the expression of heparanase and VEGF can be significantly decreased by PI-88 in OIR mice. Furthermore, PI-88 decreased the expression of heparanase in Group B and access to the expression of heparanase in Group A. Thus, PI-88 can decrease the expression of heparanase that it has increased due to oxygen induction. PI-88 is a mixture of highly sulfated oligosaccharides derived from the yeast *Pichia (Hansenula) holstii* NRRL Y-2488 and is a potent inhibitor of heparanase [13,40,41]. PI-88 shows antiangiogenic activity in vitro and in vivo, which is attributable to three known mechanisms: 1) inhibition of heparanase, an endoglycosidase that releases VEGF and active complexes of fibroblast growth factor by cleaving heparan sulfate proteoglycans in blood vessel basement membranes and the extracellular matrix; 2) direct inhibition of the binding of heparan sulfate to VEGF and fibroblast growth factor; and 3) stimulation of the release of tissue factor pathway inhibitor, an endogenous antiangiogenic protein [13,42,43]. In this study, treatment with PI-88 resulted in a decrease in the expression of VEGF and heparanase. Together, these results indicate the decreased VEGF expression may be due to the inhibition of heparanase expression by PI-88 treatment.

In conclusion, our study, using the OIR mouse model, suggested that heparanase expression is upregulated in association with VEGF expression in OIR retinas, and inhibition of heparanase expression may be a novel therapeutic strategy for ROP. In a future study, we will focus on the mechanisms of hypoxia in upstream regulatory factor for heparanase expression, and shed light on the clinical treatment of ROP.
